# Effects of a Participatory Ergonomics Intervention With Wearable Technical Measurements of Physical Workload in the Construction Industry: Cluster Randomized Controlled Trial

**DOI:** 10.2196/10272

**Published:** 2018-12-19

**Authors:** Mikkel Brandt, Pascal Madeleine, Afshin Samani, Jeppe ZN Ajslev, Markus D Jakobsen, Emil Sundstrup, Lars L Andersen

**Affiliations:** 1 National Research Centre for the Working Environment Copenhagen Denmark; 2 Sport Sciences Department of Health Science and Technology Aalborg University Aalborg Denmark

**Keywords:** back pain, low back pain, shoulder pain, musculoskeletal pain, musculoskeletal diseases, occupational health, building industry, heavy industries, organizational ergonomics, action research

## Abstract

**Background:**

Construction work frequently involves heavy physical work, and a reduction of the physical workload should have high priority. Technological development has made it possible to obtain field measurements with surface electromyography (sEMG), kinematics measured with inertial measurement units (IMUs), and video recordings. However, no studies have used these methods simultaneously to detect situations with excessive physical workload (events) during a working day. Thus, knowledge about these specific events may combat work-related risk factors. Participatory ergonomics (PE) has shown promising results, but whether it can be used as a tool to reduce the physical workload during construction work remains unknown.

**Objective:**

This cluster randomized controlled trial investigated whether a PE intervention with technical measurements consisting of IMUs, sEMG, heart rate monitoring, and video recordings of physical workload could reduce the number of events with excessive physical workload during a working day. Furthermore, other outcomes were obtained from questionnaires.

**Methods:**

A total of 80 male full-time construction workers (aged 19 to 67 years) were randomized at the cluster level (gang) to a PE intervention consisting of 3 workshops (7 gangs and 32 workers) or to a control group (8 gangs and 48 workers). The physical workload was recorded by technical measurements, that is, IMUs, sEMG, heart rate monitoring, and video recordings during a full working day at baseline and 3 and 6 months’ follow-up. On the basis of the technical measurements, a custom-made computer program detected the situations (events) where the construction workers were exposed to excessive physical workload and used in the intervention. Differences in the number of events from baseline to follow-up between intervention and control were evaluated using linear mixed models (intention-to-treat), with individual nested in cluster as a random factor. Furthermore, questionnaires were filled out on test days.

**Results:**

The results of the primary outcome showed no change in the number of events with excessive physical workload. However, compared with the control group, the other outcomes showed decreased general fatigue after a typical working day (*P*=.001) and increased influence on own work (*P*=.04).

**Conclusions:**

This PE intervention with technical measurements did not reduce the number of events with excessive physical workload during construction work. However, the intervention led to decreased general fatigue and increased influence on own work.

**Trial Registration:**

ClinicalTrials.gov NCT02498197; https://clinicaltrials.gov/ct2/show/NCT02498197 (Archived by WebCite at http://www.webcitation.org/74SZ3DIWS)

## Introduction

### Background

Work-related musculoskeletal disorders (WMSDs) such as low back pain and shoulder pain constitute a substantial problem for individuals, workplaces, and societies [1-3]. At the individual level, WMSDs increase risk of poor health, sick leave, and premature exit from the labor market [1,4,5]. For workplaces, workers with WMSDs have lower workability and are more likely to have long-term sickness absence [6,7]. For the societies, WMSDs lead to substantial expenses regarding treatment, lost production, and sickness absence [1,8]. Heavy physical work is a known risk factor for developing WMSDs [9,10]. In particular, heavy lifting, pushing or pulling, and working in awkward postures have been associated with low back pain [11] and sickness absence [4,12,13]. Construction work consists of a high degree of heavy physical work [14,15]. Consequently, a reduction of the physical workload to promote sustainable working careers [16] in construction work should have high priority. Moreover, a systematic review highlighted an urgent need for interventions focusing on reducing WMSDs in construction workers [17]. In addition, most field studies in the construction industry are based on self-reported measurements [17]. Hence, a more technical approach may enable objective evaluation of the loading and provide better grounds for targeted and effective interventions.

### Technical Measurements

Technological development has made it possible to obtain field measurements with surface electromyography (sEMG) [18,19], kinematics measured with inertial measurement units (IMU) [20-23], or a combination [24]. However, no studies have used sEMG, IMU, and video recordings obtained simultaneously to detect events with excessive physical workload (events) during a working day. Thus, knowledge about these specific events may be an important tool for engaging workers to combat work-related risk factors.

### Participatory Ergonomics

In participatory ergonomics (PE), the workers are involved in the decision processes. Systematic reviews have reported that PE has positive effects on musculoskeletal symptoms [25] and thereby may lead to increased productivity and reduced occupational risk factors [26]. Furthermore, a systematic review has shown that participatory responsibility concerning the identification of risk factors, development of solutions, and implementation is important to succeed in the participatory process [27]. Nevertheless, the evidence for preventing neck-shoulder and low back pain through ergonomics interventions is questionable because the number of randomized controlled trials are limited [28].

### Objectives

This cluster randomized controlled trial investigated whether a PE intervention with technical measurements could reduce the number of events with excessive physical workload during a working day in the construction industry We hypothesized that the PE intervention involving both managers and workers would lead to a reduction in the number of events of excessive physical workload.

## Methods

### Study Design

This study was a 2-armed, parallel group, single-blinded, cluster randomized controlled trial with allocation concealment performed at construction sites across Denmark from May 2016 to June 2017. Clusters were defined as construction gangs. The organization of construction work, that is, working in construction gangs, was the reason for choosing a cluster design. The intervention consisted of 3 workshops based on individual technical measurements of excessive physical workload. The technical measurements to detect excessive physical workload have previously been validated in controlled laboratory settings [29] and were conducted at baseline and 3 and 6 months’ follow-up.

### Ethics

According to the Helsinki declaration, participants received written and oral information about the purpose and content of the study before signing the informed consent form. The study was approved by the local ethical committee of Frederiksberg and Copenhagen (H-3-2010-062) and registered with the Danish Data Protection Agency (215-57-0074) and ClinicalTrials.gov (NCT02498197). The reporting followed the CONSORT statements for cluster randomized trials [30] and CONSORT eHealth [31] (Multimedia Appendix 1). Design of the effect evaluation and process evaluation have previously been reported [32,33]. This study reports data solely from the effect evaluation.

### Participants

**Figure 1:**
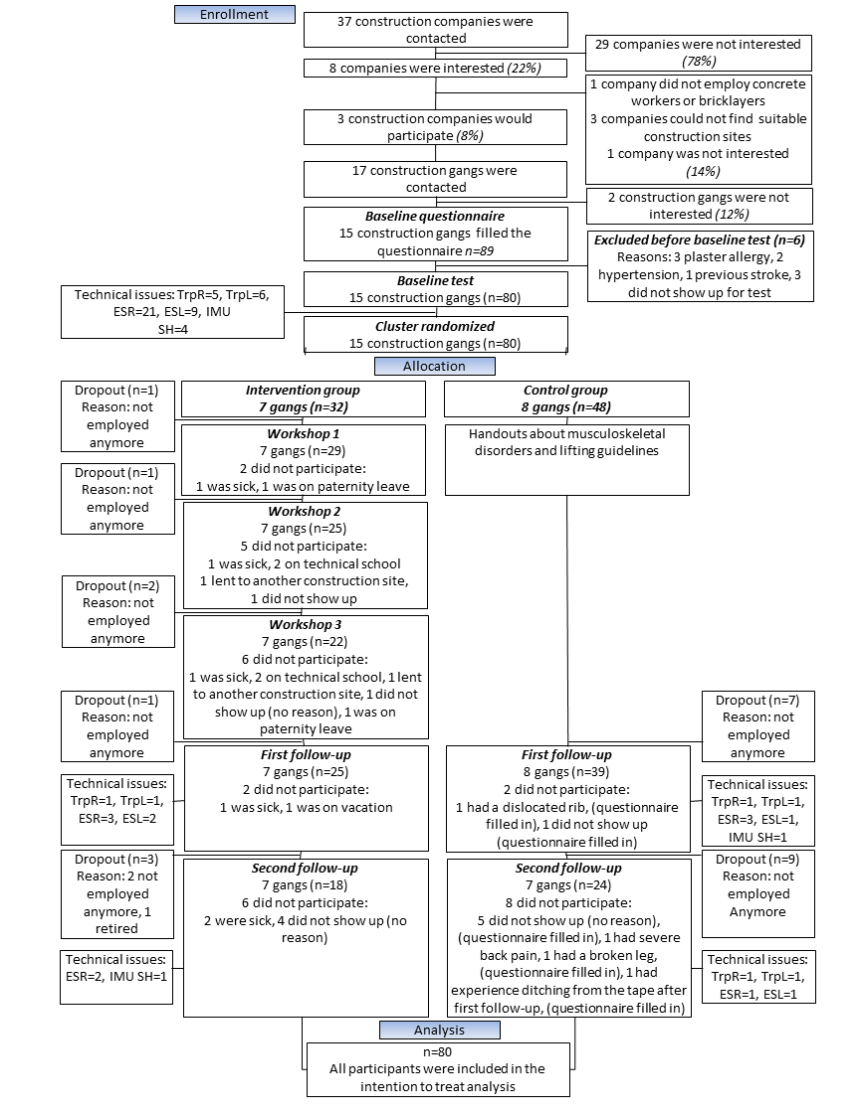
Participant’s recruitment flowchart. TrpR: trapezius right; TrpL: trapezius left; ESR: erector spinae right; ESL: erector spinae left; IMU SH: inertial measurement unit, shank.

The inclusion criterion was full-time construction work. The exclusion criteria were life-threatening diseases and hypertension >160/100 mmHg. A total of 9 participants were excluded before the baseline test. Moreover, 80 participants (15 clusters (gangs)) met the inclusion criteria and completed the baseline test. The flow of participant enrollment is illustrated in Figure 1.

### Randomization and Blinding

The randomization was performed by a researcher who was not involved in data collection (LLA). Block randomization of the construction gangs was chosen for practical reasons and was performed continuously as the baseline tests were completed. The researchers performing the data collection were not aware of the block size or group allocation. Blinding of participants is not possible in behavioral interventions. The data analyst and the statisticians were blinded to group allocation.

### Intervention

The intervention was carried out at gang level and consisted of workshops or reading handouts for the intervention group and control group, respectively.

#### Intervention Group

The workshops were organized in a 3-phase structure inspired by an action research approach [34]. The programs aimed to create possibilities for change by enabling engagement between the technical measurements and the participants. The first workshop was designed with main inspiration from the *future workshop*; a type of workshop that usually consists of 3 phases: (1) critique, (2) utopia, and (3) realization [35]. In our design, the critique phase was replaced by an introduction of the video recordings of the participants’ own work and a description of the physical workload measured in relation to each video recording. Subsequently, the participants decided which work situations should be modified during the intervention. In the *utopian phase*, the participants discussed in groups each selected work situation. The participants were to discuss and describe how the selected work process could be carried out in the best of all worlds with minimal physical exertion. In this phase, the participants were instructed not to take any barriers into account to facilitate creative resourcefulness. In the *realization phase*, the participants were asked to consider possibilities and barriers to reach the utopias. Furthermore, a plan of action was written.

In the second workshop, the participants were asked to recapture the focal points of the first workshop and to describe the progress concerning each of the selected topics in the first workshop. Then, they were encouraged to describe the barriers they had encountered in the process of reaching the goals of changing the working situations. Following this, the researchers described the current knowledge on organizational and social practices about WMSDs in the construction industry. The purpose of this was to nudge the participants to increased creativity and to challenge potentially frozen conceptions of how work should be done. Finally, the participants were encouraged to come up with further ideas on how to work toward the utopias or to aim for new utopias if they had reached their initial goals.

The third workshop had the purpose of anchoring initiatives. The researchers initially asked the participants to describe the status of the goals set earlier in the project. Then, the participants were invited to discuss whether the organization would be able to implement the initiatives of the project into long-term working practice and to come up with ideas for initiatives that could help secure this long-term anchorage.

### Control Group

The control group received handouts about WMSDs [36] and lifting guidelines from The Danish Working Environment Authority [37]. These handouts described the association between WMSDs and the impact on working life, regulations for the prevention of WMSDs, and which precautions should be taken to limit WMDSs [36]. Furthermore, the handouts described the regulations for lifting, pushing and pulling, and the risk of injuries [37].

#### Technical Measurements

At baseline and 3 and 6 months’ follow-up, the participants were equipped with sEMG, IMU, cameras, and heart rate monitors. The sEMG, IMU, and camera were synchronized [29], whereas the heart rate monitor was used to estimate the overall activity level during the working day.

The procedure for placement of sEMG electrodes is described elsewhere [29,32,38]. In short, sEMG electrodes (Blue Sensor N-00-S/25, Ambu A/S, Ballerup, Denmark) were placed bilaterally over the erector spinae and the upper trapezius muscles [39] according to the Surface Electromyography for the Non-Invasive Assessment of Muscles (SENIAM) recommendations [40]. A reference electrode was placed over the C7 vertebra. The sEMG signals were amplified 19.5 times using a 24-bit portable data-logger (Nexus10, Mind Media, Herten, Netherlands) and sampled at 1024 Hz.

IMU including triaxial accelerometer and gyroscopes (ActiGraph GT9X Link, ActiGraph, Pensacola, United States) were positioned on the upper back at T1-T2 level [41] and the thigh. The latter was used for obtaining the number of steps per day [42]. When positioned, the IMUs were calibrated in a standing neutral position (N-pose) for 15 seconds. Kinematics data were sampled at 100 Hz.

A body-worn video camera with a resolution of 848x480/30F (Reveal Media, RS2-X2L, Hampton Wick, Surrey, United Kingdom) was placed around the chest and recorded the area in front of the participant.

For heart rate monitoring electrodes (Ø: 68 mm; Blue Sensor VL-00-S/25, Ambu A/S, Ballerup, Denmark) were positioned just below the apex of the sternum and laterally under the left pectoralis major muscle [43,44], before connecting to the heart rate monitor (Actiheart, CamNtech Ltd, Cambridge, United Kingdom). Heart rate was sampled at 128 Hz and interpolated with a resolution of +/-1 ms.

The data from the IMU, sEMG, and cameras were synchronized using a custom-made device and a MatLab (2013a) program. A 2 mV trigger signal was sent to the EMG logger. At the same time, the IMUs were turned 95 degrees using a rotary solenoid (GDAX 050 X20 B71 24V, 100% ED). The synchronization with the cameras was obtained by having the cameras record a custom-made flashing device that flashed at the same time as the signal was sent to the sEMG logger box and the rotary solenoid. The synchronization was made before the equipment was positioned on the participant and repeated after the working day [29].

#### Test Protocol

The test protocol consisted of (1) maximal voluntary contractions (MVCs) for the lower back and shoulders, (2) reference lifting, and (3) calibration of the IMUs.

The MVCs for the upper trapezius was performed with a strap around the wrist in a standing position with 90-degree bilateral shoulder abduction. The participants performed maximal bilateral shoulder abduction. For the MVCs for the erector spinae, the participants were fixed with a strap around the shoulders with a slight flexion in their back leaning toward a pillow at the height of the anterior iliac spine on the hip. The participants performed 3 repetitions for each MVC with a 30 seconds rest period between the trials. The participants ramped up the force to maximum in 2 to 3 seconds and held it for 3 seconds. The participants performed 10 reference lifts from floor to table (73 cm high) with a 20 kg box (width: 56 cm, length: 34 cm, and height: 20 cm) using a forearm horizontal distance. From a starting standing position, the participants descended without load and lifted the box from the floor onto the table. After a pause of 2 seconds, they lifted the box to the floor and returned to starting position. Following a break of 2 seconds, the lifting cycle was repeated. The IMUs were calibrated by having the participants standing in a neutral position (N-pose) for 15 seconds. After these preliminary steps, the participants started their planned work and the attached equipment continuously captured data.

#### Data Analysis-Event Detection

The analyses for detecting the events are described in detail elsewhere [29]. In short, the sEMG segments corresponding to the references lifts and the MVC trials were extracted. For each of the MVC trials, the sEMG root mean square (RMS) was calculated over 500 ms epochs with 20% overlap between successive epochs. Then the maximum of calculated RMS across the epochs was found, and out of the 3 MVC trials, the highest RMS value was considered as the maximum voluntary electrical activity. Similarly, the 90th percentile of calculated RMS during the reference lift was considered as the reference threshold. Subsequently, the recorded signals during the working time were analyzed over 10-second nonoverlapping epochs. Similar to what is described above, in each epoch, the 90th percentile of the calculated EMG RMS was derived, and the extent of forward and sideways inclination of the IMU concerning the N-pose position was calculated [45]. During the working time, each of the 10-second epochs were labeled an event if the calculated sEMG amplitude was higher than the event threshold (either the average of the reference lifts in the morning and afternoon or 50% of the average MVC [46] in the morning and afternoon) for at least two of the muscles. Furthermore, for the erector spinae muscle on both sides, the event threshold was linearly decayed based on the calculated forward and sideways inclination such that the threshold would be reduced to its half at 90 degrees forward or 30 degrees sideways inclinations and it would be fixed beyond that level of inclination. The minimum of the modified threshold for the forward and sideways inclination was utilized as the modified threshold. If any of the calculated sEMG RMS over the 10-second epochs for the erector spinae on both sides was greater than the modified threshold, the 10-second epoch was labeled as an event as well. Furthermore, as exploratory analyses, the number of events was calculated based on a higher reference value of 150% of the sEMG from the reference lifts and 50% of the sEMG from MVCs. The criteria for events from the analyses were that at least two muscles should exceed the limit.

### Outcomes

#### Primary Outcome

The primary outcome was defined as the change in the number of events with excessive physical workload from baseline to follow-up. The reference lift of 20 kg used for normalization purposes was a deviation from the protocol study [32] as we planned to use 30 kg. However, because 30 kg exceeds the acceptable lifting limit of The Danish Working Environment Authority, we chose to decrease the load.

#### Other Outcomes

Other outcomes were obtained from previously established and validated questionnaires and included physical (Borg category ratio 10 scale [Borg CR10]) [47,48], psychosocial, and organizational conditions (AH2012 and COPSOQ) [49-51]. Furthermore, the pain intensity in the last week (WAS-scale) [32] was enquired.

### Sample Size

The sample size was calculated based on the observed changes in the level of muscular activity during a working day in different occupational groups with pronounced lifting [19]. The power calculation showed that 17 participants in each group were needed to demonstrate a reduction of 20% in normalized sEMG assuming an SD of approximately 20% in normalized sEMG between individuals and a type 1 risk of 5% and power of 80%. Due to the cluster design and including an inflation factor of 1.5, 26 participants were required in each group [32]. For generalizability and risk of dropouts, we aimed to recruit 10 construction gangs of 3 to 5 individuals in each group, that is, a total of 80 participants.

### Statistics

*t* tests assessed possible group differences at baseline. The difference from baseline to follow-up between the intervention and control groups was evaluated using a linear mixed model. The number of events was log-transformed because the residuals were not normally distributed. Factors included in the model were group (intervention and control), time (baseline, first follow-up, and second follow-up), and *group-by-time* interaction. The analysis was adjusted for the baseline value of the outcome, age, gender, duration of measuring time, mean heart rate, number of steps, and muscle strength. Individual nested in cluster was included as a random factor. Analyses were performed using SAS statistical software (Proc Mixed, SAS version 9.4) according to the *intention-to-treat* principle, including all participants (n=80) regardless of loss to follow-up. The estimation method was restricted maximum likelihood with degrees of freedom based on the Kenward-Roger approximation. *P* levels ≤.05 were accepted as statistically signiﬁcant. Outcomes are reported as within- and between-group least square mean differences with 95% CIs. Furthermore, Fischer exact test was used to test for differences in questions with categorical response variables.

## Results

### Participant Characteristics

Table 1 shows the baseline characteristics of the participants. Age was higher in the control group compared with the intervention group (*P*=.02), which was controlled for in the statistics by including age as a covariate. At the first follow-up test, 12 participants dropped out, and 4 participants did not show up for the test. At the second follow-up test, 12 participants dropped out, and 14 participants did not show up for the test. Hence, 42 participants completed the study (Figure 1). All dropouts were included in analyses.

### Primary Outcome

The results showed no *group-by-time* interaction effect (*P*=.75) and (*P*=.51) for the number of events obtained using technical equipment in the unadjusted and adjusted analysis, respectively (Table 2). The results show a within-group difference (*time* effect) in the number of events from baseline to the first follow-up test (unadjusted, *P*=.002 and adjusted *P*=.05 and (unadjusted *P* **<**.001 and adjusted *P*<.001) for the intervention and control group, respectively. Furthermore, a within-group difference was observed from baseline to second follow-up in the unadjusted analysis for the intervention and control group (*P*<.01 and *P*<.001), respectively. The exploratory analyses confirmed the results of the primary outcome, that is, no significant *group-by-time* interaction (Table 2).

The analyses of heart rate and step count showed no *group-by-time* interaction or within-group difference. However a between-group difference was observed at baseline, first and second follow-up, and at baseline for heart rate (*P*<.001, *P*=.049, and *P*=.003, respectively). This between-group difference was also observed for the step count at baseline (*P*=.004) but not at the follow-ups. The mean heart rate was 100 (95% CI 96 to 104), 101 (95% CI 97 to 105), and 100 (95% CI 96 to 105) and 91 (95% CI 88 to 95), 95 (95% CI 92 to 99), and 91 (95% CI 87 to 95) bpm for the intervention and control group at baseline, first follow-up, and second follow-up, respectively. The mean number of steps adjusted for length of the working day were 5952 (95% CI 5517 to 6387), 5479 (95% CI 5023 to 5934), and 5980 (95% CI 5372 to 6588) and 5133 (95% CI 4791 to 5475), 5340 (95% CI 4958 to 5722), and 5320 (95% CI 4852 to 5788) steps per day for the intervention and control group at baseline, first follow-up, and second follow-up, respectively.

### Other Outcomes

The results from the other outcomes are presented in Tables 3 and 4. In the intervention group, the results showed a within-group decrease in general fatigue after a typical working day (*P*=.001; Table 3) from baseline to second follow-up and in influence on own work from baseline to first follow-up (*P*=.04; Table 3). The remainder of the other outcomes showed no effect from the intervention (Tables 3 and 4).

### Adverse Events

No adverse events were reported.

**Table 1:** Basic characteristics of the participants in the study.

Characteristics			Intervention group		Control group
Number of participants, n (all males)			32		48
Age in years, mean (SD)			34.2^a^ (12.5)		41.2^a^ (12.5)
Height in centimeters, mean (SD)			180.0 (6.2)		180.1 (7.2)
Weight in kilograms, mean (SD)			85.0 (12.2)		86.4 (14.6)
Weekly working hours, mean (SD)			39.1 (4.8)		38.1 (2.5)
Gang size, mean (SD)			4.5 (1.5)		6.0 (2.3)
**Smokers, n (%)**					
	Yes	15 (47)		17 (36)	
	No	17 (53)		31 (64)	
**Current position, n (%)**					
	Concrete workers	25 (78)		27 (56)	
	Bricklayers	5 (16)		19 (40)	
	Others (eg, bricklayer´s assistant)	2 (6)		2 (4)	
**Term of employment, n (%)**					
	Hourly paid	13 (41)		35 (73)	
	Monthly paid	1 (3)		0 (0)	
	Paid according to performance	18 (56)		13 (27)	
**Experience in construction, n (%)**					
	<3 years	4 (12)		4 (8)	
	4-10 years	14 (44)		13 (27)	
	>11 years	14 (44)		31 (65)	
**How often can you take it easy and still reach your working tasks?, n (%)**					
	Always	0 (0)		1 (2)	
	Often	6 (19)		11 (23)	
	Sometimes	16 (50)		23 (48)	
	Rarely	9 (28)		12 (25)	
	Never	1 (3)		1 (2)	
**How exhausting do you find your regular work? (Borg CR10^b^), n (%)**					
	Light (0-2.5)	1 (3)		6 (12.5)	
	Moderate (3-5)	16 (50)		36 (75)	
	Hard (6-10)	15 (47)		6 (12.5)	
**How often do you feel pain in your body? n (%)**					
	Every day	10 (31)		16 (33)	
	A few times a week	10 (31)		8 (17)	
	A few times a month	8 (25)		18 (38)	
	Maximum a few times a year	4 (13)		6 (12)	
	Never	0 (0)		0 (0)	
**Degree of difficulty in the low back within the last week (0-10 VAS^c^), n (%)**					
	0-3	13 (42)		24 (52)	
	4-6	10 (32)		15 (33)	
	7-10	8 (26)		7 (15)	
**Degree of difficulty in the upper back within the last week (0-10 VAS^c^), n (%)**					
	0-3	19 (61)		35 (76)	
	4-6	8 (26)		9 (20)	
	7-10	4 (13)		2 (4)	
**Degree of difficulty in the shoulders within the last week (0-10 VAS^c^), n (%)**					
	0-3	23 (74)		32 (70)	
	4-6	6 (19)		11 (24)	
	7-10	2 (6)		3 (6)	

^a^Difference between groups at baseline, *P*=.02.

^b^Borg CR10: Borg category ratio 10 scale.

^c^VAS: visual analog scale.

**Table 2:** Results of the primary outcome (change from baseline to follow-up in events with excessive physical workload during a working day) from the mixed-model analysis.

Group and outcome				Within-group difference					Between-group difference at follow-up			
Group			Baseline	First follow-up	*P* value	Second follow-up	*P* value	First follow-up		*P* value	Second follow-up	*P* value
**Primary outcome**												
	**100% sEMG^a^ from reference lifts-unadjusted (95% CI)**											
		Intervention	5.2 (5 to 5.5)	5.8 (5.5 to 6.1)	.002	5.8 (5.4 to 6.1)	.01	−0.1 (−0.4 to 0.3)		.72	0 (−0.4 to 0.4)	.94
		Control	5.1 (4.9 to 5.3)	5.9 (5.6 to 6.1)	<.001	5.8 (5.5 to 6)	<.001	—^b^		—	—	—
	**100% sEMG from reference lifts-adjusted (95% CI)**											
		Intervention	5.4 (5.1 to 5.7)	5.8 (5.5 to 6.1)	.05	5.7 (5.3 to 6.0)	.3	0 (−0.4 to 0.4)		.89	0.1 (−0.4 to 0.6)	.62
		Control	5.1 (4.9 to 5.4)	5.8 (5.6 to 6.1)	<.001	5.5 (5.2 to 5.9)	.052	—		—	—	—
**Explorative analysis**												
	**150% sEMG from reference lifts (95% CI)**											
		Intervention	3.8 (3.5 to 4.2)	4.3 (3.9 to 4.8)	.06	3.8 (3.3 to 4.3)	.94	0.3 (−0.2 to 0.8)		.21	0.4 (−0.2 to 0.9)	.89
		Control	3.6 (3.2 to 3.9)	4.0 (3.6 to 4.4)	.08	3.9 (3.4 to 4.3)	.3	—		—	—	—
	**50% sEMG from MVCs^c^ (95% CI)**											
		Intervention	4.2 (3.7 to 4.8)	4.2 (3.6 to 4.9)	.97	4.8 (3.9 to 5.6)	.35	0.2 (−0.6 to 1.1)		.6	0.6 (−0.5 to 1.7)	.3
		Control	4.0 (3.5 to 4.4)	4.0 (3.4 to 4.6)	.89	4.2 (3.4 to 4.9)	.62	—		—	—	—

^a^sEMG: surface electromyography.

^b^Not applicable.

^c^MVCs: maximal voluntary contraction.

**Table 3:** Results from the other outcome.

Group and scale		Baseline	First follow-up	Second follow-up	Between-group difference at follow-up		Time effect, *P* value	
					First follow-up	Second follow-up		
**Heaviest lift last week, 0-10 scale (95% CI)**								
	Intervention	6.7 (6.1 to 7.2)	6.8 (6.2 to 7.4)	6 (5.3 to 6.7)	0.2 (0.6 to 0.1)	−0.1 (−1 to 0.8)	.52	
	Control	6.2 (5.7 to 6.6)	6.5 (6 to 7)	6.1 (5.5 to 6.6)	—^a^	—	—	
**General fatigue after a typical working day, 5-point scale (not tired, a little tired, tired, very tired, and exhausted) converted to 0-100 (95% CI)**								
	Intervention	41.1 (37.2 to 45)	40.8 (36.6 to 45)	35.6 (31 to 40.3)	−6.1 (−11.7 to −0.5)	−11.2 (−17.4 to −5)	.001	
	Control	39.4 (36.2 to 42.5)	46.9 (43.4 to 50.4)	46.8 (42.9 to 50.7)	—	—	—	
**How physically strenuous do you usually perceive your current work?, Borg CR10 scale^b^ (95% CI)**								
	Intervention	4.8 (4.2 to 5.3)	4.4 (3.9 to 5)	5 (4.4 to 5.7)	−0.4 (−1.1 to 0.4)	0.5 (−0.3 to 1.4)	.10	
	Control	4.3 (3.9 to 4.8)	4.8 (4.3 to 5.3)	4.5 (3.9 to 5)	—	—	—	
**How much influence do you have on your work, 5-point scale (very much, much, some, little, very little) converted to 0-100 (95% CI)**								
	Intervention	59.6 (56.6 to 62.5)	60.4 (57.3 to 63.6)	58.9 (55.4 to 62.5)	5.6 (1.5 to 9.8)	−0.1 (−4.8 to 4.6)	.04	
	Control	60.3 (58 to 62.7)	54.8 (52.2 to 57.4)	59 (56 to 62)	—	—	—	
**Do you wish more influence on your work, 2-point scale (yes or no) converted to 0-100 (95% CI)**								
	Intervention	39.3 (29.6 to 49)	49.4 (39 to 59.7)	48.7 (37.3 to 60.2)	−3.9 (−17.6 to 9.7)	0.1 (−15.3 to 15.4)	.85	
	Control	44.1 (36.3 to 51.9)	53.3 (44.7 to 61.9)	48.7 (38.9 to 58.5)	—	—	—	

^a^Not applicable.

^b^Borg CR10: Borg category ratio 10 scale.

**Table 4:** Results from the other outcome. Numbers indicate the participants who answered the question (percent of the population who answered the question).

Group and question		Baseline					First follow-up					Second follow-up				
		Daily	Weekly	Hardly ever	*P* value^a^	Daily		Weekly	Hardly ever	*P* value^a^	Daily		Weekly	Hardly ever	*P* value^a^	
**How often do you perform heavy lifting?, n (%)**																
	Intervention	21 (66)	11 (34)	0 (0)	.2	14 (50)		13 (46)	1 (4)	.54	11 (50)		11 (50)	0 (0)	.22	
	Control	23 (48)	24 (50)	1 (2)	—^b^	19 (48)		16 (40)	5 (12)	—	10 (32)		18 (58)	3 (10)	—	
**How often do you feel pain in your body (eg, arms, hands, knees, shoulders, and back)?, n (%)**																
	Intervention	10 (31)	10 (31)	12 (38)	.32	8 (29)		7 (25)	13 (46)	.53	3 (14)		10 (45)	9 (41)	.59	
	Control	16 (33)	8 (17)	24 (50)	—	14 (34)		13 (33)	13 (33)	—	8 (26)		12 (39)	11 (35)	—	
**Do you take analgesics because of pain in your neck/shoulders or back?, n (%)**																
	Intervention	1 (3)	1 (3)	30 (94)	.65	1 (4)		2 (7)	25 (89)	.86	0 (0)		1 (5)	21 (95)	.37	
	Control	3 (6)	4 (8)	41 (86)	—	2 (5)		5 (13)	33 (82)	—	2 (6)		4 (13)	25 (81)	—	

^a^Between group differences.

^b^Not applicable.

## Discussion

### Principal Findings

This study is the first to detect events with excessive physical workload using only technical measurements, individual thresholds, and applying these measurements in a PE intervention. The results of this cluster randomized controlled trial showed that a PE intervention did not decrease the number of events during a working day. Other outcomes showed positive effects on influence on own work and general fatigue after a typical working day, but not on pain, perceived workload, and how often heavy lifting was performed.

### Interpretation of Results

Technical measurements have the advantage of being objective. Furthermore, it builds on from standardized analytical procedures of the raw data, rather than, for example, self-reports or visual observations [52,53]. We have recently shown that the intraday reliability for sEMG during lifting tasks is acceptable in laboratory conditions [38]. Furthermore, the method for detecting events of the lower back and shoulder based on sEMG and IMU from the upper back has shown high accuracy in a laboratory setting [29]. Thus, we are certain the measurement method per se was not cause of the nonsignificant findings.

There was no *group-by-time* interaction for the primary outcome, which was the change in the number of events from baseline to follow-up. However, the number of events increased over time in both groups, and because this technical detection was used for the first time in a field study, we performed exploratory analyses with the detection of events based on 150% of the sEMG obtained during the reference lifts and 50% MVCs (Table 2). This confirmed that there was no effect of the intervention on the primary outcome. However, the exploratory analysis did not show a within-group difference as seen in the preplanned analysis [32]. This could be related to the threshold, which might have been too low, and we might have seen a more stable normalization factor by using, for example, 30 kg as reference value. However, the within-group increase in both groups could also be related to the organization of construction work, which is characterized by a distinctive variation regarding work pace, work tasks, and work processes. This variation makes it challenging to conduct intervention studies with field measurements in the construction industry as the inherent variance will necessitate a larger sample size than anticipated based on laboratory measurements. As the participants were further into the process of their current construction project during the follow-up than at baseline, this may have increased the work pace due to incentive reasons. However, analyses of heart rate and step count did not support this speculation. Another possibility could be that the participants were more aware of the measurements at baseline and therefore acting more carefully to avoid heavy lifting. This effect may have diminished during follow-up.

The other outcomes showed effects on influence at work and general fatigue after a typical working day. The difference in influence on own work was only seen at first follow-up and was primarily driven by the control group experiencing a decrease in influence. This may indicate that the control group felt neglected compared with the intervention group who attended the workshops and had the opportunity to bring forward new ideas. The second follow-up was 3 months after the last workshop. Therefore, the feeling of being neglected might have eased off, likely because the tangible consequences of the intervention were often only “increased attention to physically stressful work” as shown in Multimedia Appendix 2, rather than real changes in the working process or technical assistive devices. The decrease in general fatigue after a typical working day in the intervention group indicates that some effect occurred in response to the intervention despite not being effective in reducing events. It can be speculated that the implemented solutions led to work that reduced light loads, repetitive work, or made the work processes more efficient in general and thus less physically fatiguing. A review has shown reductions in physical work demands and musculoskeletal symptoms if mechanical lifting devices are introduced at workplaces [54], and other studies have shown a decreased discomfort [55] or ergonomic improvement when introducing devices for raised bricklaying that may decrease the physical workload during construction work [56]. As the majority of the implemented suggestions concerned technical assistive devices, it can be speculated that the increased use of assistive devices may partially explain the decrease in general fatigue.

The majority of the suggestions were related to assistive devices (Multimedia Appendix 2) and are in accordance with previous findings, where the workers identified ergonomic solutions using assistive devices to reduce WMSDs, but the support from the contractors to implement these was lacking [57]. Accordingly, other studies suggest that support from the management is critical for providing changes in the construction industry [58,59]. In this study, the management was often not willing to support the suggestions if they involved increased costs. Hence, more support from the management might have had a positive effect [60]. The intervention might have failed in involving the management as we underestimated the challenge of obtaining economical and persistent commitment from the management. However, this seems to be a highly common but underaddressed issue in participatory research [27,61].

There may be several contributing factors to the high physical demands of construction work, of which work organization plays an important role. Construction work in Denmark is characterized by being organized in small working units, often on a group-based wage, which can be associated with an increased risk factor for WMSDs [62] and can induce a group pressure within the gang to get the work done at a certain time without taking pain into consideration [63]. Studies suggest that both structural and cultural changes are necessary to create changes in the construction industry [57,64]. The lack of effect from the intervention in this study might be related to the culture in the construction industry where WMSDs are an accepted part of being a construction worker [65,66].

### Perspectives

With the rapid technological development, this method could be integrated into portable devices connected to, for example, mobile phones and thereby provide the worker with direct feedback to prevent work tasks with excessive physical workload.

### Strengths and Limitations

A strength of this study is the cluster randomized design, making it possible to intervene at gang level, thus reducing the risk of a number of biases associated with nonrandomized studies. However, there are also known challenges of conducting behavioral randomized controlled trials, for example, blinding of participants or potential participants and supervisors who do not accept randomization [67]. Another strength is the use of technical measurements to quantify the workload rather than relying on self-reports or observations.

A limitation of this study is that the number of dropouts was higher than expected, resulting in reduced statistical power. The terms of employment in the construction industry are dominated by short-term contracts, which resulted in a relatively high turnover of workers in the participating gangs in this study. This affected the number of participants employed over the entire intervention period. In research involving randomized controlled trial, it is preferable to have a stable group of participants. However, to our knowledge, no participants drop out of the study due to a lack of willingness to participate but were missing at random, and all participant were included in the intention-to-treat analysis. To fully control a randomized controlled trial in the construction industry showed to be extremely difficult due to, for example, variation in work tasks performed during a working day and sudden changes based on unpredictable incidents at the construction site. On the other hand, a considerable strength of this study is that the measurements have been conducted during actual construction work. Furthermore, it is a limitation that the size of the individual clusters was larger than anticipated, which also reduced the statistical power due to intracluster correlation. In general, the variance in measurements was also higher than expected. Due to these factors, future studies would need to recruit a much larger sample size to be randomized. On the other hand, the results of this study do not indicate that a relevant between-group difference would be reached even with a larger sample size.

The loss of data from sEMG, especially from the erector spinae muscles at baseline, was also a limitation. This loss of sEMG data was primarily caused by electrodes that slipped off, and future studies should minimize this loss of data by securing the sEMG cables such that excessive sweating of the participants does not compromise the skin-electrode impedance. Larger band aids over the electrodes or performing the measurements during the cooler season of the year when sweating is not a big issue might also help.

The inherent variation in daily working tasks at the construction sites is a practical challenge because the necessary sample size can easily grow to a level that is not realistic to achieve. We tried to control this by having close contact with the construction sites and conducting measures on the workers during similar working tasks, but this was not always possible. However, we compensated for this by controlling for steps and heart rate in the analysis.

During the recruitment, we were in contact with many small-scale construction companies that were unable to participate because their job tasks did not permit the long follow-up time in this study. Hence, we only included large-scale construction companies; thus, one should be cautious about generalization of our results to small-scale construction companies. On the other hand, changes are often even more difficult to implement in smaller companies where resources are scarce. It is, therefore, unlikely that inclusion of smaller companies would have changed the main conclusion of the study.

Finally, the difference in age and, partly, experience between the intervention and control group could be limitations to the study. Therefore, we controlled for age in the statistical analysis, but it cannot be ruled out that a more experienced intervention group could have increased the implementation rate of the suggested solution and thus reduced the number of events with excessive physical workload following the intervention.

### Conclusions

This PE intervention with 3 workshops did not reduce the number of events with excessive physical workload during construction work. An exploratory analysis using higher thresholds confirmed the results. The intervention group experienced a reduced general fatigue and an increased influence on own work following the intervention, compared with the control group.

## Appendix

Multimedia Appendix 1 CONSORT‐EHEALTH checklist (V 1.6.1).

Multimedia Appendix 2 Descriptions of the workshops, including issues and solution from the participants. OHS: occupational health consultants, OHC: occupational health chief, int: internal, ext: external.

## Abbreviations

IMU: inertial measurement units
MVC: maximal voluntary contraction
PE: participatory ergonomics
RMS: root mean square
sEMG: surface electromyography
WMSD: work-related musculoskeletal disorder
