# Supplementary material for: Effects of a Participatory Ergonomics Intervention With Wearable Technical Measurements of Physical Workload in the Construction Industry: Cluster Randomized Controlled Trial
Source: J Med Internet Res. 2018 Dec 19;20(12):e10272. doi: 10.2196/10272 (PMC6315250; doi:10.2196/10272)
Supplement: Multimedia Appendix 2 [file jmir_v20i12e10272_app3.pdf]

		Workshop 1			Workshop 2		Workshop 3		
Construction gang		Issues	Participants	Suggested solution	Participants	Suggested solution	Participants	Suggested solution	Implemented actions
No. 1	Concrete workers	Formwork	5 Workers	Light weight beams	8 Workers	Warm up session before starting the work	9 Workers	Better planning	Increased attention to physically stressful work
	n=6	Heavy lifting	2 OHS	Light weight molds	2 OHS	Focus on lifting technique	2 OHS		
		Work from ladders	1 Foreman						
No. 2	Concrete workers	Heavy lifting	5 Workers	Use lifts instead of ladders	With gang 1		With gang 1		Increased attention to physically stressful work
	n=5	Work from ladders	1 OHS	Two person to carry beams					
		Element assemble		Formwork assemble area					
No. 5	Concrete workers	Heavy lifting	5 Workers	Rebar binder	4 Workers	Evaluation of action plan	4 Workers	Evaluation of action plan	Rebar binder (binding in small dimensions)
	n=5	Reinforcement and binding		Include the foreman in planning	1 Foreman		1 Foreman		
No. 6	Concrete workers	Formwork	6 workers	Use the crane more	4 Workers	Possibility for lighter concrete forms	4 Workers	Evaluation of action plan	Increased attention to physically stressful work
	n=6	Concrete casting	1 Foreman	Job rotation when using concrete vibrator	1 Foreman	Smaller concrete vibrators tested	1 Foreman		
		Concrete vibration		Focus on lifting technique	2 OHS				
				Smaller concrete vibrators	*(1 int 1 ext)*				
No. 7	Concrete workers	Heavy lifting	3 Workers	Focus on lifting technique	3 workers	Evaluation of action plan	1 Worker	Evaluation of action plan	Increased attention to physically stressful work
	n=3	Reinforcement and binding	1 Foreman (partly)	Raise the height of the iron					
		Handling of iron							
No. 3	Bricklayers	Handling mortar and stones	3 Workers	New brick splitter	1 worker	Windshield	2 Workers		New brick splitter
	n=3	Too high bricklaying	1 OHC	Increase the height of the brick splitter		Toolbox on the working floor			Raised working height on brick splitter
		Heavy lifting				Change the height of the scaffolding			Windshield
No. 4	Bricklayers	Heavy lifting	3 Workers	Use Manitou more	4 workers	Use Manitou more	4 Workers	Electric trolley for bricks	New brick splitter
	n=4	Cutting bricks	1 OHS	Less load on the wheelbarrow			1 OHC	Raised storage for bricks	Increased attention to physically stressful work
		Too high bricklaying	1 Foreman	New brick splitter					
